# A comprehensive situation assessment of injection practices in primary health care hospitals in Bangladesh

**DOI:** 10.1186/1471-2458-11-779

**Published:** 2011-10-10

**Authors:** AK Azad Chowdhury, Tapash Roy, ABM Faroque, Sitesh C Bachar, Muhammad Asaduzzaman, Nishat Nasrin, Nahid Akter, Hamidur Rahman Gazi, Abul Kalam Lutful Kabir, Masuma Parvin, Claire Anderson

**Affiliations:** 1 Department of Clinical Pharmacy & Pharmacology, University of Dhaka, Bangladesh; 2 Division of Social Research in Medicine and Health, School of Pharmacy, University of Nottingham, UK

## Abstract

**Background:**

Understanding injection practices is crucial for evidence-based development of intervention initiatives. This study explored the extent of injection use and injection safety practices in primary care hospitals in Bangladesh.

**Methods:**

The study employed both quantitative and qualitative research methods. The methods used were - a retrospective audit of prescriptions (n = 4320), focus group discussions (six with 43 participants), in-depth interviews (n = 38) with a range service providers, and systematic observation of the activities of injection providers (n = 120), waste handlers (n = 48) and hospital facilities (n = 24). Quantitative and qualitative data were assessed with statistical and thematic analysis, respectively, and then combined.

**Results:**

As many as 78% of our study sample (n = 4230) received an injection. The most commonly prescribed injections (n = 3354) including antibiotics (78.3%), IV fluids (38.6%), analgesics/pain killers (29.4%), vitamins (26.7%), and anti-histamines (18.5%). Further, 43.7% (n = 1145) of the prescribed antibiotics (n = 2626) were given to treat diarrhea and 42.3% (n = 600) of IV fluids (n = 1295) were used to manage general weakness conditions. Nearly one-third (29.8%; n = 36/120) of injection providers reported needle-stick injuries in the last 6 months with highest incidences in Rajshahi division followed by Dhaka division. Disposal of injection needles, syringes and other materials was not done properly in 83.5% (n = 20/24) of the facilities. Health providers' safety concerns were not addressed properly; only 23% (n = 28/120) of the health providers and 4.2% (n = 2/48) of the waste handlers were fully immunized against Hepatitis B virus. Moreover, 73% (n = 87/120) of the injection providers and 90% (n = 43/48) of the waste handlers were not trained in injection safety practices and infection prevention. Qualitative data further confirmed that both providers and patients preferred injections, believing that they provide quick relief. The doctors' perceived injection use as their prescribing norm that enabled them to prove their professional credibility and to remain popular in a competitive health care market. Additionally, persistent pressure from hospital administration to use up injections before their expiry dates also influenced doctors to prescribe injections regardless of actual indications.

**Conclusions:**

As far as the patients and providers' safety is concerned, this study demonstrated a need for further research exploring the dynamics of injection use and safety in Bangladesh. In a context where a high level of injection use and unsafe practices were reported, immediate prevention initiatives need to be operated through continued intervention efforts and health providers' training in primary care hospitals in Bangladesh.

## Background

Unsafe injection practices have an inherent risk of spreading three preventable primary blood borne viral (BBV) pathogens; human immunodeficiency virus (HIV), hepatitis B virus (HBV) and hepatitis C virus (HCV) [1,2]. Preference of injections to oral medications and widespread misuse of injections in many developing countries has long been of great concern to health professionals and the World Health Organization, but so far little systematic research has been conducted into this world-wide practice. Available information suggests that the use of injections in developing countries is common and often unnecessary [3,4].

Further, unsafe injection practices, such as reuse of contaminated needles and syringes in many parts of the world, are also common [5-7]. In developing countries, the estimated number of injections per person per year is 3.4 (range 1.7-11.3) and the proportion of unsafe injections is estimated to be 39% (range 1.2-75%) [3]. In some countries of South Asia the estimate is as high as 75% [3]. A number of studies in India and Pakistan have identified unsafe injection practices [5,6,8,9], and BBV infections have been attributed to unsafe injection practices [9-11].

Recently, a small-scale study conducted in few primary health care hospitals in Bangladesh has found very high rates of misuse and unsafe practices. The study estimated that some 60% - 80% of used injections were either unnecessary or avoidable - e.g. antibiotic injections were used to treat diarrhea or antihistamine injections used for skin disease [12]. Such practices are clearly exposing a huge number of patients to the risk of BBV infections [7]. In addition, it drains scarce financial resources from both the private and public sectors. Very limited information is available to provide an evidence-based understanding on the national picture of injection practices in Bangladesh.

A recent intervention study using monitoring-training-planning (MTP) strategy (an intervention which combines managerial, educational and regulatory strategies)[4] showed that the intervention has reduced the use injections in some health complexes in Bangladesh [13]. The intervention results are only indicative rather than confirmatory owing to lack of proper expertise, trained personnel, funding and sound methodology; and a small sample size in limited study areas. Therefore, a well designed study was needed in order to provide an evidence-based understanding of injection practices in Bangladesh and to develop appropriate interventions to promote safe injection practices with an aim to reduce the associated risk of BBV infections that are transmitted through unsafe injection procedures [3,10,14].

The current study assessed injection practices in selected Upazila Health Complex (UHC) hospitals from all six geographical divisions in Bangladesh. It was a fundamental need in a country where a high prevalence of HBV (5.5%-10%) and HCV (2.8%-3.6%) have been documented among general population [15-19]. The threat of HIV infection is also looming in Bangladesh, as the country is geographically surrounded by high HIV prevalence neighboring states of India and also by Myanmar with high rates of cross-border migration [20]. Epidemiologically, Bangladesh is currently moving towards a 'concentrated HIV epidemic' state with an increasing prevalence of HIV (7.1%) among injecting drug users in Dhaka [21]. Misuse and unsafe injection practices may further facilitate epidemic spread of these BBV infections. The purpose of this study was: to examine the extent to which injections were used in UHCs in Bangladesh, the indications for which they were given, the type and degree of improper and unsafe practices in the process of administration of injections, the way in which they were perceived, and the extent to which facilities in the study areas meet necessary requirements for practices, equipment and waste disposal procedures. An additional objective was the development of an intervention to promote injection safety practices.

## Methods

### Study design

An observational study was designed which employed both quantitative and qualitative research methods. The study was conducted between May 2008 and February 2009 and consisted of three components: i) a retrospective audit of prescriptions to determine injection use patterns; ii) systematic observation of UHCs facilities, injection providers, waste handlers, and injection administering events to assess how injection safety measures were followed; and iii) six focus group discussions (FGDs) with doctors and 38 in-depth interviews (IDIs) with doctors, nurses, and waste handlers on injection safety practices. Qualitative data supplemented the quantitative findings for practices that were not identified in the survey.

### Study sites

This study took place in 24 primary health care centers (known as Upazila Health Complexes) of six divisions in Bangladesh. Geographically Bangladesh is divided into six divisions (namely Dhaka, Chittagong, Khulna, Rajshahi, Sylhet and Barisal). We randomly selected four Upazila Health Complexes (UHCs) from each of these geographical divisions. The UHCs are government health facilities located at sub-district level and offer mostly primary (in limited areas provide secondary level care) health care. Each UHC serves an estimated population of 0.5 million in rural Bangladesh. We excluded district level (which provides secondary level care) and Medical College Hospitals (provides tertiary level care) from this study, as our focus of interest was to assess injection practices in primary health care settings.

### Data collection tools

The questionnaire for prescription audit and injection safety observation tools were developed based on previous research tools [12,22,23], which have been modified in accordance with the World Health Organization (WHO)/Safe Injection Global Network (SIGN) recommended revised Injection Safety Assessment Tool [24].

In addition to socio-demographic characteristics, the survey instrument included information on injection prescribing pattern and safety practices. The indicators included; total number of prescribed medications per patient, total number of injections used per prescription, percentage of prescribed injections per prescription, injection use rate (percentage of patients who received one or more injection), percentage of injection used in certain tracer health conditions (e.g. diarrhea, skin disease, fever), percentage of injection prescribed in specific health conditions, frequency distribution of types of injections (e.g. antibiotics, intravenous fluids, pain killers/analgesics, anti-histamines) given per tracer condition.

As outlined in WHO/SIGN revised Injection Safety Assessment Tool [24], injection safety practices were observed under four sub-categories, such as indicators for: i) health facilities and general safety for heath workers, recipients and community (e.g. proportion of facilities where injection safety or waste management policy/guidelines were available for viewing, percentage of facilities with no overflowing or pierced sharps containers, percentage of facilities with no used sharps in an open container or open areas, proportion of facilities with safe final waste disposal methods); ii) injection providers' safety (e.g. percentage of providers: reported needle stick injuries in the last six months, who were trained in injection safety practices, who were immunized against hepatitis B); iii) patients/injection recipients safety (e.g. percentage of facilities with no used sharps in open areas, percentage of events providers maintained antiseptic procedure, percentage of events where a new syringe- needles were used every time, percentage of recipients who reported a adverse event, percentage of adverse events followed up after injection procedures); and iv) safety of waste handlers (e.g. percentage of waste handlers: have had access to and used 'heavy protective gloves', received formal training in healthcare waste management in the last two years, fully immunized for hepatitis B, and reported needle stick injury in the last six months).

To facilitate data collection for interviews and focus groups two separate topic guides were developed for IDIs and FGDs respectively. The guides covered a range of issues like providers' perspectives on injection uses; justifications of injection uses; causes of unsafe injection practices; risk perceptions; present knowledge and practices on injection safety (they included knowledge about risk of needle stick injury, diseases likely to be transmitted, individuals at risk etc.); syringes and needles disposal; types of syringes available; monitoring and regulatory mechanism related to safe injection use; and barrier to safe injection practices.

The draft data collection tools for all components of the study were piloted in one health facility in Dhaka, which resulted in a number of modifications. The final version was written in English and then translated into Bengali. Finally, the Bengali version was back-translated into English to check for linguistic validity of the tools.

### Definitions

For the purpose of this study we considered injections as any medications that were injected either intravenously or intramuscularly or subcutaneously; we also included intravenous (IV) fluid administration.

The injection use rate in a facility was defined as the percentage of prescriptions at a certain health facility that list at least one or more injections. It was expressed as: Number of prescriptions examined during the study period containing at least one or more injections/Total number of prescriptions examined in a certain facility × 100. The injection use rate per prescription was defined as the percentage of injection prescribed per patient's prescription.

Safe/unsafe injection: An injection was considered safe if it did not harm the recipient, did not expose the provider to any avoidable risks and did not result in waste that is dangerous for the providers, recipients and community [25,4,23,24]. On the contrary, an injection was therefore considered unsafe if it harmed the recipient, exposed the provider to any avoidable risks and resulted in waste that was dangerous for the providers, recipients and community.

Injection safety was defined as practices that intended to prevent transmission of infectious diseases between one patient and another, or between a patient and healthcare provider, and also to prevent harms such as needle-stick injuries, and to ensure safe environment for providers, patients and community through appropriate management of dangerous medical waste [23,24].

### Sampling and data collection

A research team consisting of eight trained research assistants (postgraduate pharmacy and medical students) collected survey and observation data of the study. Monitoring and supervision was provided by the principal investigators (AKAC and CA) and other members of the investigation team (co-investigators). The interviews and focus groups data were collected by co-investigators with supports from the research assistants.

### Gaining access to the research sites

In order to gain access to the research sites, we acquired written permission from the Director General of Health Services as per the requirement of the Ministry of Health, Bangladesh. At local level, further discussions were held between the research team and the local hospital administrators of each site in order to facilitate data collection process. Based on our initial discussions, the hospital authorities of each site organized group information sessions with the representatives from all categories of service providers, where we informed them about the research, and the nature and process of data collection. A written information sheet outlining the nature of the research and the process of data collection was also circulated during information sessions. During specific types of data collection, for example systematic observations, providers who were being observed were informed about the research and the nature of the observations on a one-on-one basis and finally informed written consent was taken from each of them.

### Study component I: retrospective audit of prescriptions on injection use

**Figure 1:**
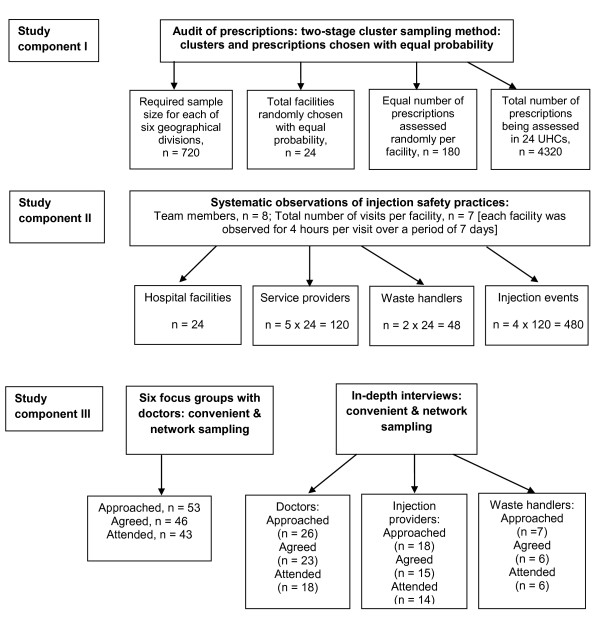
**Flow of participates through the study (sampling & recruitment)**.

We collected retrospective prescribing data on injection use from hospital records. For sample size calculation, we assumed an alpha error of 0.05, precision of 5%, a design effect of 2 and considered prevalence of injection use as unknown. Considering these assumptions and including 15% of missing or incomplete data and the need to adjust for possible confounders, it was determined that a sample size of 708 from each division was required for the study. To round up and to cover further short-fall, we considered 720 prescriptions from each division, which yielded a total sample size of 4320 for the audit (Figure 1).

A two-stage, cluster-sampling method was used for the sample size calculation. From the whole country, six geographic divisions were selected with a probability proportional to population size. As illustrated in Figure 1, location clusters (24 UHCs) were chosen randomly at the first stage with equal probability with an aim to enroll 4 facilities from each division (4 × 6 = 24) on the basis of accessibility, non-ongoing interventions and maximum scattering across the country. A fixed number of prescriptions (n = 180) was then chosen from each of the selected clusters (each UHC) with equal probability based on available list of the patient records (prescribed between January 2006 and January 2008) and using random number tables.

### Study component II: systematic observation of injection safety practices

Prospective data on injection safety practices were collected through observation visits to each of the selected facilities. The research team members observed each health facility for 4 hours per day over a period of seven days. The team observed the activities of 120 injection providers, 48 waste handlers and 24 hospital facilities to access how safety measures were followed and to look for evidence of injection practices. A total of 672 hours of observation (28 hours per facility) took place in a range of clinical areas characterized by frequent use of needles and other sharps, and waste disposal areas. Injection providers' and waste handlers' activities were observed on the spot based on their availability during the period of observation visits. We also observed a total of 480 injections being administered by 120 injection providers.

During each visit injection safety practices were observed in terms of injection preparation, administration of injection to the patients and disposal of syringe/medical waste in order to give a clear understanding of whether the health providers followed standard guidelines or not [22,23].

### Study component III: in-depth interviews and focus group discussions with a range of providers

This phase of the study involved in-depth interviews (IDIs) with the doctors, nurses and waste handlers, and FGDs with doctors of 24-UHCs to explore issues that were unanswered from the audit and/or observation data, and to supplement the findings of each other components. Potential participants for IDIs and FGDs were recruited either through convenience or network sampling techniques during audit or observation visit to the facilities. A total of 38 semi-structured interviews with doctors (n = 18), nurses/medical assistants (n = 14) and waste handlers (n = 6) of UHCs and six FGDs (with 5-7 doctors in each group, n = 43) were conducted in the local language. All in-depth interviews and focus groups were audio recoded.

Informed consent was requested from each participant and those who agreed were interviewed. The study protocol was reviewed and approved by the experts and review committees at the University of Dhaka, Bangladesh and the University of Nottingham, United Kingdom.

### Data analysis

All statistical analyses were conducted using SPSS version 15 (SPSS Inc., Chicago). Calculations of frequencies, proportions, and χ^2 ^tests were used to assess the significance of relationships between outcomes and explanatory indicators.

All qualitative data sources (interviews, focus groups, field observation notes) were assembled and interview tapes were translated and transcribed verbatim. After becoming familiar (that involves reading and re-reading of transcript texts, reviewing notes) with the content of texts, we identified a thematic framework that enabled us to recognize emerging themes or issues in the data set [26]. Extensive discussions were held between the members of researcher team to explore the key themes. On the basis of these discussions, we developed coding matrices for thematic analysis based on Ritchie and Spencer's framework approach [26] and entered data from all sources into these. As far as the approach is concerned, during the analysis stage we sifted, charted and sorted gathered data in accordance with key issues and themes. We then identified sections of the data that correspond to a particular theme and placed the specific pieces of indexed data in charts that consist of the headings and subheadings (drawn during the thematic framework). Finally, we mapped and interpreted the analysis of the key characteristics as laid out in the charts [26]. This rigorous process allowed us to identify key themes, explore discourses, and compare these across data and/or respondents, and to generate new ideas where appropriate. Results were then compared and discrepancies were discussed with the wider group, and concepts were further refined.

Finally, qualitative data was organized, and the central themes/findings were constantly compared and combined with results of quantitative data to see how findings complemented each other. The key findings from both data sets were then analyzed in relation to wider perspectives of injection uses and injection safety practices (locally and where applicable internationally).

## Results

### The extent of injection use

Table 1 shows the extent of injection use patterns in six divisions of Bangladesh. Injections were given in more than three quarter (77.7%, n = 3354) of the studied illness cases in the health facilities. Among the six divisions, the injection use rates were the highest in the UHCs of Chittagong division (94.2%) and lowest in the UHCs of Dhaka division (60.2%). The average number of injections per patient per prescription was 2.7 (range 1.0 - 4.3). This was also the lowest in UHCs of Dhaka division (average 1.0) and the highest in UHCs of Khulna division (average 4.3).

**Table 1:** The extent of injection use patterns in selected UHCs of six divisions in Bangladesh

Facilities	Injection use rate (%)	No. of injection/person (SD)
**Dhaka Division (n = 720)**	**60.14**	**1.04 (± 0.03)**
Mirzapur UHC (n = 180)	70.39	1.47 (± 0.05)
Kaliakoir UHC (n = 180)	61.90	1.07 (± 0.02)
Gazaria UHC (n = 180)	57.80	0.98 (± 0.01)
Bondor UHC (n = 180)	50.47	0.62 (± 0.05)
**Chittagong Division (n = 720)**	94.18*	**3.92 (± 0.12)**
Anoara UHC (n = 180)	98.33	5.72 (± 0.16)
Sitakunda UHC (n = 180)	93.89	2.33 (± 0.09)
Manikchari UHC (n = 180)	92.83	3.30 (± 0.11)
Rangunia UHC (n = 180)	91.67	4.34 (± 0.14)
**Rajshahi Division (n = 720)**	**70.92**	**2.26 (± 0.08)**
Durgapur UHC (n = 180)	76.99	2.80 (± 0.10)
Bagmara UHC (n = 180)	74.19	2.67 (± 0.09)
Godagari UHC (n = 180)	67.50	1.69 (± 0.06)
Puthia UHC (n = 180)	65.00	1.88 (± 0.07)
**Khulna Division (n = 720)**	**82.92**	**4.26 (± 0.13**)
Tala UHC (n = 180)	95.00	6.75 (± 0.18)
Avoynagar UHC (n = 180)	90.00	5.48 (± 0.16)
Batiaghata UHC (n = 180)	69.44	2.36 (± 0.09)
Fultala UHC (n = 180)	77.22	2.45 (± 0.09)
Batiaghata UHC (n = 180)	69.44	2.36 (± 0.09)
**Sylhet Division (n = 720)**	**82.36**	**2.15 (± 0.08)**
Chatak UHC (n = 180)	96.67	2.40 (± 0.09)
Fenchugonj UHC (n = 180)	86.66	2.70 (± 0.10)
Bianibazar UHC (n = 180)	73.33	1.60 (± 0.06)
Golapgonj UHC (n = 180)	72.77	1.90 (± 0.07)
**Barisal Division (n = 720)**	**75.42**	**2.32 (± 0.76)**
Gournadi UHC (n = 180)	93.89	2.56 (± 0.09)
Uzirpur UHC (n = 180)	81.67	3.27 (± 0.11)
Nalchiti UHC (n = 180)	79.44	2.27 (± 0.08)
Babugonj UHC (n = 180)	46.66	1.16 (± 0.03)

**Total (n = 4320)**	**77.65**	**2.66 (± 0.08)**

UHC, upazila health complex; SD, standard deviation; *p < 0.01

As shown in Table 2, the most commonly used injections included antibiotics (78.3%), IV fluids (38.6%), analgesics/pain killers (29.4%), vitamins (26.7%), and anti-histamines (18.5%). The injections were frequently used to treat acute watery diarrhea (36.7%), general weakness (19.4%), skin infections/itching (15.4%), fever (13.6%) and traumatic injuries (11.2%).

**Table 2:** Types and patterns of injection use

Variables	Injection use rate (%)
**Percentage of injection use vs**. **other medications (n = 4230)**	
Injections only	68.7
Oral medications only	20.8
Injections and oral medications	10.2
Other medications	1.3
Types of Medications (n = 3354)*	
Antibiotics	78.3
Intravenous fluids	38.6
Analgesic/pain killers	29.4
Vitamins	26.7
Anti-histamines	18.5
Sedatives	13.4
Anti-malarial	08.6
Others	24.8
**Number of injections per prescription (n = 3354)**	
1-2	32.8
> 2	67.2

*Adds up to more than 100% because analysis is derived from the analysis of multiple responses using Statistical Package for the Social Sciences.

Table 3 further investigates indications for which specific categories of injections were given. Among 2626 cases where antibiotics were prescribed, 43.7% (n = 1145) was prescribed to treat acute watery diarrhea. Further, vitamins (91.7%) and anti-histamines (73.6%) injections were frequently used for general weakness and skin infections respectively. Where IV fluids were used (n = 1295), 42.3% (n = 600) of them were used to manage non-specific condition such as general weakness.

**Table 3:** Types of injection used vs. health conditions

Health conditions/Types of injection used	Antibiotics (N = 2626)	IV fluids (N = 1295)	Analgesics/pain killers (N = 986)	Vitamins (N = 896)	Anti-histamines (N = 620)
	
	% (n)	% (n)	% (n)	% (n)	% (n)
Acute Diarrhea	43.7 (1145)	31.4 (407)	-	-	-
General weakness	-	42.3 (600)	-	91.7 (822)	5.7 (35)
Skin infection/itching	23.5 (617)	4.8 (62)	5.2 (51)	-	73.6 (456)
Fever	22.7 (596)	14.5 (188)	63.4 (625)	3.6 (32)	9.2 (57)
Traumatic injury/assault/RTA	13.2 (347)	3.4 (44)	22.7 (224)	2.8 (25)	8.5 (53)

RTA, road traffic accident; IV, intravenous

### Injection safety practices

Table 4 demonstrates injection safety practices in the UHCs of Bangladesh. In general, none of the health facilities (n = 24) observed had injection safety and waste disposal policy or guidelines available for viewing. Immediate disposal of used needles and syringes in a puncture proof sharps container or use of a needle remover was not observed in more than two-third (81.5%) of the health facilities. Only in 16.5% of all facilities (n = 24) syringes and needles were disposed of in a puncture proof container and of those 55% facilities in Dhaka, 36.3% in Chittagong and 20% in Rajshahi division used puncture proof containers for sharp materials disposal. None of the facilities in Sylhet, Barisal and Khulna division had any puncture proof containers at all to dispose of sharp materials. We observed that overflowing sharps container were found in 78% of the UHCs and in 80.7% of the facilities there were loose injection materials scattered around the open areas. Only 16.2% of the facilities were found with safe final waste disposal methods. Further, during observations we found that used syringes and intravenous fluid bags (drips) were scattered around, not only at health facility waste sites but also inside the hospital buildings (including consultation areas and wards) as well. In as many as 89.2% sites we found that children were playing with used syringes and intravenous fluid bags.

**Table 4:** Division-wise injection safety practices in the UHCs in Bangladesh

Indicators	Division§						Total
		
	1	2	3	4	5	6	
**A. Facility observation: general safety (n = 24)**							
Average no. of injections per patient (number)	0.9	3.8	02.3	02.3	02.31	04.3	2.7
Injection safety policy/guidelines was available for viewing (%)	0.0	0.0	0.0	0.0	0.0	0.0	0.0
Waste disposal policy/guidelines was available for viewing (%)	0.0	0.0	0.0	0.0	0.0	0.0	0.0
Waste handlers have access to 'heavy gloves' (%)	0.0	0.0	0.0	0.0	0.0	0.0	0.0
Facilities with no non-sharps infectious waste outside (%)	23.5	16.3	11.6	0.0	0.0	0.0	8.6
Used syringe, needle disposed in puncture proof box (%)	55.0*	36.3	20.0	0.0	0.0	0.0	16.5
Facilities with no loose sharps/needles overflowing (%)	36.5	48.3*	47.5	0.0	0.0	0.0	22.0
Facilities with no loose injection materials scattered (%)	23.8	40.0*	26.5	0.0	11.2	15	19.4
Facilities with safe final waste disposal methods (%)	31.5	12.5	21.5	10.12	0.0	21.3	16.2
							
**B. Injection providers' safety (n = 120)**							
Providers reported needle stick injuries in last 6 months (%)	46.3	32.5	67.5*	0.0	32.5	0.0	29.8
Providers trained in injection safety practices in last 2 years (%)	87.5*	0.0	75.0	0.0	0.0	0.0	27.1
Providers have had at least one Hepatitis B vaccination	3.6	27.3	13.7	53.4	47.6	0.0	24.3
Immunized against HBV [all 3 doses] (%)	02.5	25.0	12.5	52.5*	45.0	0.0	22.9
							
**C. Safety of the recipients (n = 480)**							
Providers washed hands with antiseptic soap/rub (%)	33.5*	0.0	0.0	0.0	0.0	0.0	05.6
Patients whose injection site sterilized by spirit (%)	100.0*	0.0	08.8	30.0	20.0	12.5	28.5
Used new syringe- needles for reconstitution (%)	98.0	88.5	80.7	74.5	83.0	82.5	84.5
Injection was prepared on clean table (%)	56.5	0.0	05.0	50.0	80.0*	18.8	35.0
Used syringe, needle disposed immediately after injection	55.0*	36.3	20.0	0.0	0.0	0.0	18.5
No used sharps were scattered in open areas	27.8	36.0*	22.5	4.5	11.2	14.7	18.8
Followed adverse events after injection (%)	13.8	0.0	0.0	0.0	30.0*	0.0	07.3
							
**D. Safety of the waste handlers (n = 48)**							
Waste handlers used 'heavy gloves' (%)	0.0	0.0	0.0	0.0	0.0	0.0	0.0
Trained in healthcare waste management (%)	50.0*	0.0	0.0	12.5	0.0	0.0	10.4
Immunized with HBV vaccine [3 dose] (%)	9.7	6.5	0.0	08.8	0.0	0.0	4.2
Waste handlers used any form of gloves (%)	62.5*	0.0	0.0	0.0	0.0	0.0	10.4
Reported needle stick injury in last 6 months (%)	46.5*	20.5	27.5	16.3	30.6	10.8	25.4

HBV, hepatitis B virus; §1 = Dhaka, 2 = Chittagong, 3 = Rajshahi, 4 = Sylhet, 5 = Barishal, 6 = Khulna.

* p < 0.01

Further, providers were not trained in injection safety practices and medical waste management. It was noted that 87.5% of the injection providers (n = 120) in the health facilities of Dhaka division and 75% in Rajshahi division have received some sort of training in injection safety practices, but none of the providers in the UHCs of all other study sites in Khulna, Chittagong, Barisal and Sylhet division were trained. Similarly only 50% of the waste handlers (n = 48) in Dhaka division and 12.5% in Sylhet division were trained in healthcare waste management. Moreover, in all observed facilities none of the waste handlers have had access to "heavy protective gloves".

We also observed a total of 480 injection events being administered by 120 injection providers (4 events per provider) to look at how safe the processes were for the injection recipients and also for the injection providers in real practice. Findings demonstrated that none of the injection providers washed their hands properly with antiseptic soap or used alcohol based rub in UHCs of Chittagong, Rajshahi, Khulna, Sylhet and Barisal division. The only exception was observed in Dhaka division where 33.5% providers did so. In contrast, when we asked individually during interviews almost all providers reported that they have washed their hands properly with antiseptic soap or used alcohol based rub.

All service providers (100%) in UHCs of Dhaka division cleaned or wiped the injection site with rectified spirit before providing injections, but this practice was very poor in all other divisions (ranged from 8.8% in Rajshahi division to 30% in Sylhet division). In 84.5% of all injections being administered (n = 480), the injection providers used new syringes and new needles. However, in 15.5% events we observed the providers reused the same syringes and needles up to three times (specifically for very poor patients). The unsafe practices of reusing contaminated needles and syringes were higher in UHCs of Sylhet Division (25.5%) than other divisions (between 2.0% and 17.5%). Further, in 35% of all injection events (n = 480), the injection providers did not always prepare the injections on a clean table or tray.

Needle-stick injuries were common with 67.5% of the injection providers in Rajshahi, 46.3% in Dhaka and 32.5% in Chittagong and Barisal reporting having had such injuries in the last six months before the survey. This could be a potential source of transmission of BBV infections from the infected patients to the injection providers. On the other hand, none reported such injuries in Khulna and Sylhet division. On further investigation concerning the safety of the health workers, we found that only 23% of injection providers (n = 120) and 4.2% of the waste handlers from all study sites were fully immunized against hepatitis B virus (all 3 doses completed).

### Contextual factors contributing to popularity of injections and unsafe injection practices

Qualitative data also revealed that injections were prescribed to most of the patients. Most doctors reported treating 50-60 patients per day (including patients in their private practice). In most of cases injection were prescribed and diagnosis were made based on only clinical presentation of the cases. They were rarely supported by any laboratory investigations. Hence providers often have made decisions for prescribing antibiotic injections based on their clinical judgments and perceived seriousness of the diseases. During in-depth interviews and FGDs with doctors and health assistants the respondents mentioned that their perception of the seriousness of the disease may not be the correct one as they were hardly ever supported by standardized, evidence-based treatment protocols for the treatment of common illnesses.

The reason behind the popularity of injections was fairly complex and is summarized below.

Disease conditions where injections were frequently used

• Watery diarrhea/dehydration

• General weakness

• Skin infections

• Fever/pyrexia of unknown origin

• Road traffic accidents and assault cases

• Reparatory infection/pneumonia

• Abdominal pain/peptic ulcer

Reasons behind popularity of injections

• Local beliefs about illness and concepts of efficacy

• Perceived severity of the disease (doctors perspective):

- Doctors' belief that injections are more effective than oral medications

- Perceived seriousness of the diseases by the doctors often motivated them to use injection

- A perception that serious conditions need serious and "powerful medication"

• Doctors' own quest to prove their superiority over other doctors through prescribing so called high-cost "powerful medications".

• Lack of patient-provider communication.

• The sensitivity of the patients' condition

• Patients' demand

- A desire to get quick relief: mostly derived from the perceptions that injections are the sure way to quick relief

- A perception that injections are necessary in a good prescription

- A notion to justify strong police cases against the offenders (in case accident/assault incidences)

• Pressure from hospital authority

- To use up the injections before their expiry dates

- To release in patients from the hospitals as quickly as possibly

(affecting doctors prescribing behavior to treat patients more aggressively with injections)

• Prescribing and dispensing by the medical assistants/paramedics

- A tendency to over-prescribe injections

- Prolonged absence of doctors from hospitals

- Emergency case management in absence of doctors

• Economic interests of pharmaceutical companies

- Aggressive marketing/promotional activities

- Attractive gifts offer to the doctors and paramedics/medical assistants

In both in-depth interviews and FGDs we identified multiple reasons that shaped the final decision to use injections by the doctors/medical assistants. Doctors as well as patients believed that injections work better than oral medications. In many cases injection use was predominantly influenced by doctors' beliefs that injections are more effective than oral medications and their perception of the seriousness of the diseases often motivated them to use injections. Doctors' own quest to prove their superiority over other doctors in general practice has significantly shaped their prescribing behavior and instigated them to use high-cost "powerful medications" often with an aim for magical cure. In a few cases the social status of patients also influenced doctors to prescribe injections, for example, if the patient was from an influential family in the community, doctors prescribed them injections to show that the patient was being taken care of seriously.

In many cases patients' demands also strongly influenced prescribing behavior. Patients' demand mostly emanated from their desire to get quick relief and/or a notion to justify stronger police cases against offenders (in accident/assault cases, the persons responsible for the offences). During in-depth interviews with doctors and providers it was highlighted that such demands from patients were mostly derived from the perceptions that injections are the sure way to quick relief. Doctors further mentioned that patients believed that injections are necessary in a good prescription. Hence a prescription without injections was not considered as a good prescription. In few cases an intention for better compensation and/or litigation aspects to justify documentary seriousness of the incidents like assault motivated patients to demand for injections.

Further, pressure from the health/hospital administration to use up the injections before their expiry dates, influenced the doctors to prescribe injections regardless of actual indications. During focus groups the doctors mentioned that there was also a persistent pressure from the hospital administration to release inpatients from the hospitals as quickly as possible to clear the beds for allocating them to new patients. Such pressure often affected doctors' prescribing behavior making them treat patients more aggressively with injections to attempt to cure them as quickly as possible.

It was revealed that in the absence of doctors medical assistants were required to take responsibility for handling patients and prescribing in many cases. The doctors highlighted that the medical assistants tended to overuse injections. These practices were common as some of the doctors remained absent from the health complexes for long periods of time.

It was also noted that aggressive marketing or promotional activities of injection products by the medical representatives of pharmaceutical companies have a significant influence on the prescribing behavior of the doctors. In many cases, the pharmaceutical companies offered very attractive gifts to the doctors and paramedics to prescribe their medicines. Such aggressive marketing techniques have had a significant influence on overuse of injections by the doctors.

## Discussion

In our audit of prescriptions, high rates of injection use were observed in the primary care hospitals in Bangladesh. More than three quarters of the total prescribed medications were injections and the majority (78.4%) of prescribed injections was antibiotics followed by IV fluids. The results further indicated that this high rate of injection use cannot be bio-medically justified. Antibiotics were frequently used to treat conditions like diarrhea and IV fluids, and even to manage non-specific conditions like general weakness. Further, high rates of injection use even in such uncomplicated, non-severe and self-limiting illnesses, indicating medical inappropriateness of injection use. Previous research in other developing country settings suggests that the misuse of injections remains high due to the false assumption of prescribers that injections will improve patient satisfaction and that they are always expected by the patients [27-29]. Supporting the findings from previous research in other settings, our research illustrated that both prescribers and the patients consider the inclusion of injections in a prescription as standard practice [4,6,8,9,27].

The injection use pattern was almost the same in most of the facilities studied except in the UHCs of Dhaka division, which was lower than other divisions. The injection safety practices indicators were also comparatively better in Dhaka. This may be due to the fact that there had been a few previous interventions implemented to reduce misuse and promote safe injection practices in the selected UHCs of Dhaka [12,13]. The authors of the study with the funding support from the WHO/SIGN project implemented MTP intervention in 2004/2005 in 4 UHCs (these 4 UGCs were also included in our current study) of Dhaka. It is interesting that the impact of previous interventions appear to have remained positive for some of the safe injection practice indicators in those facilitates in Dhaka division. This indicates a need to introduce similar interventions in other UHCs in Bangladesh. Such interventions should combine management, educational and regulatory strategies [4] and ensure health providers' safety, for example, they should include training in safe injection practices for both injection providers and waste handlers, improve supportive supervision of health facilities and the processes aiming to improve safe injection practices should be regularly monitored [12,13,29].

Consistent with findings from other settings, our observations suggests that injections were often not provided in safe and hygienic ways [4,6,8,29-31]. Sharps injuries happened frequently among injection providers and medical waste handlers. Surprisingly, a higher level of training of the health workers was not translated into safer injections practices. For example, the facilities in Dhaka were those most likely to dispose of used needles in a puncture-proof box, the most likely to have safe final waste disposal methods, and it was the only division where most of the staff had received training in injection safety, but they reported the second highest proportion of injection providers with needle stick injuries, and the highest proportion of waste handlers with needle stick injuries. Although we do not have any insight into why some of the other sites reported no needle stick injuries amongst the injection providers and waste handlers despite poor practices, qualitative data give us some explanations about this. During our in-depth interviews the injection providers in Dhaka frequently mentioned that the patient turn over in Dhaka is higher than other areas. As a result providers in these facilities have to work in a very busy environment and always rush to provide services, which make them more prone to accidental incidents like needle stick injuries as reported. They may also be more likely to report needle stick injuries because they have been trained.

Further, providers were not aware of the risks associated with unsafe injection practices. A lack of awareness on the part of health care workers about the risk of BBV diseases transmission in health care settings even with or without reuse of non-sterile equipment may explain a number of practices allowing for cross contamination that expose both injection providers' and recipients to infections. There was no culture to promote self-protection or to train providers in terms of safety practices. In addition, there were shortages in supply of disposable syringes and needles and other essential equipment required to practice injection procedure safely and hygienically. Most of the time patients need to buy their own disposable syringes from a private pharmacy. Poor patients, those who were unable to buy their own disposable syringes and needles, were most frequent victims of syringes and needles reuse (injection provider often administered injection to them using syringes and needles that had been used for other patients before). In the Bangladeshi setting where HBV and HCV infection have a high endemic profile and where injections are misused and/or overused, such breaks in infection prevention practices may be sufficient to transmit BBV infections from patient to patient through injections. Poor immunization against HBV and lack of training in injection safety and waste disposal has further precipitated the risk of infection [12,29].

From the view point of safety of the patients, health providers, waste handlers and the community, the injection practices were unsafe in all of the study facilities in terms of many indicators. Moreover, puncture-proof containers and heavy protective gloves for waste handlers were lacking, and medical waste was not disposed of properly. Thus, the risk of needle-stick injuries is increased, hence increasing the risk of BBV infections transmission through contaminated needles. Similar findings were reported in previous studies in different settings [30,31]. The presence of sharp waste in the environment also indicates that Bangladesh's medical waste management infrastructure needs to be strengthened.

In our in-depth interviews and FGDs, most doctors and medical assistants reported that patients, particularly older persons in rural areas, prefer injections for common medical conditions. This finding is consistent with result of other studies in different settings [9,13,27,28]. However, a number of elements suggested that health care workers significantly contribute to injection misuse or overuse. These include: a tendency of physicians' to provide rationalized and "scientific" explanations to justify their misuse/overuse; economic incentives from pharmaceutical companies; limitation of standardized and evidence-based treatment protocols for common illnesses, safety practices and medical waste management; and the persistence of inappropriate policies and pressure from hospital authorities. Echoing with the results from other studies our findings suggest that practitioners think patients want injections and this demand prompts them to administer more injections than they deem necessary to remain popular in a competitive health care market [4,9,27,28]. As a result, patients think that far more injections are required than are actually needed. A clear patient-provider communication gap exists in this issue.

The data presented here reflects the present situation in injection practices in primary care hospitals in Bangladesh. At this stage, we do not know what is ideal or the gold standard. No data are available regarding the association between the misuse of injections and infections in Bangladesh or elsewhere. Given that high levels of avoidable injection use in Bangladeshi hospitals, the study demonstrates a need for further research to explore the dynamics of injection safety practices and relationships between injections and resulting infections in different hospital contexts, and suggests a need for prevention efforts to promote safe and rational injection use in primary care hospitals in Bangladesh.

A lack of concern for the management of health care waste associated with an absence of a waste treatment infrastructure may result in the presence of sharps waste in the environment. Supportive supervision for health workers will strengthen safe injection practices and related waste management. Policy makers and implementers should focus on prevention initiatives in order to promote and ensure safe injection use and practices in primary care hospitals in Bangladesh.

### Limitations of the study

While the current study contributes important information likely to aid in the design of relevant policies, and to guide future research and programmatic efforts relevant to injection practices in Bangladesh or elsewhere, it must be considered in light of a number of study limitations. We acknowledge the general limitations of using retrospective data. The use of retrospective information rather than prospective longitudinal data for our prescription audit may limit the generalization of our findings.

Further limitations include the inability to generalize our result to other parts of Bangladesh because it is probable that given the variability of injection practices within districts (as demonstrated by our study), there is also likely to be considerable and unpredictable variability between districts. Data presented in our analyses were based on only the service provider's perspective of injections use; therefore, this may not essentially represent the patient's perspective of injections use. For example, claims were made by health care workers that patients preferred injections, but the patients were not actually interviewed or included as participants in IDIs or FGDs; this could add richness and complexity to the findings of future studies. The fact that the injection providers and waste handlers knew they were being observed may have influenced their injection practices, but this is most likely to have influenced them to practice more carefully and safely rather than less.

### Strengths of the study

Despite these limitations, our study has some strong grounds to justify the validity of the results. Our data were drawn from a mixed-method study involving a retrospective audit of prescriptions, systematic observation of injection safety practices, and FGDs and in-depth interviewers with a range of hospital workers. We consider this triangulation of data sources as a potential strength of our study, which enhanced the validity of the findings. The in-depth interviews and focus groups data supplemented the survey and observation findings (and vice versa), thus providing richer interpretation to the results. Our systematic observations also helped to contextualize the interview and focus group data and allowed the findings to go beyond the subjective perceptions of the interview participants. On the other hand, the interviews and focus groups gave depth to the observations and survey results by providing the doctors, injection providers and waste handlers with an opportunity to describe and interpret their experiences in relation to injection use and safety, and to share their views regarding reasons for misuse/overuse of injections and unsafe practices.

## Conclusion

As far as the patients and providers' safety is concerned, this study demonstrated a need for further research exploring the dynamics of injection use and safety practices in Bangladesh. In a context where a high level of injection use and unsafe practices were reported, immediate prevention initiatives need to be operated in order to promote safe injection practices in primary care hospitals in Bangladesh. Such an intervention should include components like, i) establishing clear rules and regulations for the use of injections in medical practice and promoting evidence-based practice; ii) ensuring appropriate logistics (e.g. supply of disposables and protective gloves) and safeguard procedures for injection providers and waste handlers; iii) improving supervision of health facilities and monitoring the process aiming to improve injection safety practices on regular basis; iv) making health personnel aware of the negative impact of their injection practice on the spread of BBV; v) proper training of health care providers in safe injection practices and safe management of sharps medical waste through carrying out clean injection program for providers, including in-service training, refresher courses, and guidelines; and vi) also carrying out clean injection program for users, including information, education and communication (IEC) about the need for hygienic practices.

Such interventions will only be successful if they consider the underlying perspectives of injection misuse both for service providers and for users. Therefore, any policy to be adopted must be based upon good understanding of the cultural meaning of injections and best available evidence, their place in medical practices, and their influence upon human relations.

## Competing interests

The authors declare that they have no competing interests.

## Authors' contributions

AKAC and CA conceptualized and designed the study. TR conducted the statistical and thematic analyses. TR and AKAC drafted the manuscript and incorporated all suggestions from other authors. All authors made significant contributions to the conception and design of the analyses, interpretation of the data, and drafting of the manuscript, and all authors approved the final manuscript.

## Pre-publication history

The pre-publication history for this paper can be accessed here:

http://www.biomedcentral.com/1471-2458/11/779/prepub
